# Visual Strategies of Avoidantly Attached Individuals: Attachment Avoidance and Gaze Behavior in Deceptive Interactions

**DOI:** 10.3390/jemr19010005

**Published:** 2026-01-07

**Authors:** Petra Hypšová, Martin Seitl, Stanislav Popelka

**Affiliations:** 1 Department of Psychology, Palacký University Olomouc, Křížkovského 10, 779 00 Olomouc, Czech Republic; petra.hypsova@upol.cz; 2 Department of Geoinformatics, Palacký University Olomouc, 17. listopadu 50, 771 46 Olomouc, Czech Republic; stanislav.popelka@upol.cz

**Keywords:** attachment avoidance, eye-fixations, eye-tracking, deception detection, gaze behavior

## Abstract

Gaze behavior is a critical component of social interaction, reflecting emotional recognition and social regulation. While previous research has emphasized either situational influences (e.g., deception) or stable individual differences (e.g., attachment avoidance) on gaze patterns, studies exploring how these factors interact to shape gaze behavior in interpersonal contexts remain scarce. In this vein, the aim of the present study was to experimentally determine whether the gaze direction of individuals differs, with respect to their avoidant orientation, under changing situational conditions, including truthful and deceptive communication towards a counterpart. Using a within-person experimental design and the eye-tracking methodology, 31 participants took part in both rehearsed and spontaneous truth-telling and lie-telling tasks. Consistent with expectations, higher attachment avoidance was associated with significantly fewer fixations on emotionally expressive facial regions (e.g., mouth, jaw), and non-significant but visually consistent increases in fixations on the upper face (e.g., eyes) and background. These findings indicate that stable dispositional tendencies, rather than situational demands such as deception, predominantly shape gaze allocation during interpersonal interactions. They further provide a foundation for future investigations into the dynamic interplay between personality and situational context in interactive communicative settings.

## 1. Introduction

Gaze behavior during dyadic and group interactions contributes to accurate emotional recognition [1] and, consequently, to the quality of communication and social regulation [2]. Patterns of a counterpart’s face-scanning may capture changes in all areas with critical cues [3] or reflect suboptimal procedures and thus distorted perception. Recent advances in eye-tracking technology have created new opportunities for studying these patterns of gaze behavior as a behavioral outcome of cognitive load, emotional control, and social monitoring [4,5,6,7,8,9,10,11].

However, two streams of research conclusions have emerged. The first emphasizes situational factors shaping eye movements, with significant focus on demanding situations such as shifts between truthful and deceptive communication [12,13,14,15,16]. The second highlights the systematic influence of stable individual differences grounded in personality theories [17], with considerable interest in attachment avoidance [18,19]. Both research streams are influential and independent, so conclusions about the interaction between situational factors and individual differences in shaping gaze behavior patterns are scarce. Although recent empirical studies [20,21] have identified the effects of both situational context and general individual differences on gaze behavior, and the influential theoretical review [22] proposed a framework that includes personality in explaining gaze behavior in face-to-face communication, research examining situational demands and theoretically grounded individual differences remains limited. Therefore, the aim of the present study is to experimentally determine whether individuals’ gaze direction toward regions of interest significant for emotion decoding differs according to their avoidant orientation under changing situational conditions, including truthful and deceptive communication towards a counterpart.

## 2. Theoretical Background

A particularly relevant framework for understanding individual differences in behavior during social interactions provides attachment theory [23,24], specifically the dimension of attachment avoidance. Avoidantly attached individuals deactivate their attachment system [25], distancing themselves emotionally and cognitively from distressing stimuli [26]. Unlike anxiety, which is characterized by overt hyperactivation and visible distress, avoidance involves behavioral suppression and disengagement, concealing observable indicators of underlying psychological states [27,28].

Because gaze is one of the most immediate and socially meaningful behavioral signals [12], it may offer a significant indicator for examining how avoidant individuals regulate attention in social situations. Consistent with the vigilance–avoidance hypothesis, avoidant individuals typically show an initial orientation toward emotionally salient or threatening facial regions, followed by rapid attentional disengagement as a means of emotional regulation [29,30]. This early vigilance enables quick detection of potential threat cues and facilitates the deployment of regulatory strategies to minimize emotional arousal [31]. For example, Gu et al. [31] demonstrated that although avoidant individuals initially attend to emotional facial expressions, they subsequently shift their gaze toward less emotionally informative facial regions. Similarly, Szymanska et al. [30] found that avoidant adolescents were slower to fixate on comforting images and spent less time exploring them than secure or anxious peers. This suggests that gaze serves as both a regulatory tool and a potential index of attachment-related defensive strategy to filter out specific information [32,33]. Uccula et al. [34] supported these assumptions and found that avoidant individuals showed early attention to emotional cues but later preferred neutral or irrelevant regions, consistent with a strategy of perceptual disengagement. Recent findings by Török-Suri et al. [35] further illustrated this temporal attentional dynamics: avoidant participants initially fixated longer on emotionally expressive facial regions but disengaged during later recall, reflecting a regulatory shift from monitoring to avoidance once emotional salience was detected.

The above studies support the concept of “areas of comfort” [1], which describes a general fast-scanning pattern for detecting crucial signs of emotional expression, followed by a return of gaze to a region of interest (ROI) that is individually comfortable. However, a growing body of research notes that the “area of comfort” for individuals with avoidant orientation is not simply neutral or emotionally irrelevant. In contrast to previous conclusions that disengagement increases during avoidant attention [31,34], Wu and Shimizu [19] found that avoidance is associated with greater attention to the eye-region than is typical for attachment anxiety. This means that individuals with an avoidant orientation focus more on critical cues important for recognizing anger, fear, and, to some extent, sadness, rather than on cues important for disgust or happiness [3], which are scanned only briefly. In this vein, Seitl et al. [25] found that individuals with avoidant orientation are less expressive to happy situations but not to situations involving anger. Possibly, the upper face (i.e., eye-region) may also represent an “avoidant” area of comfort, conveying a more permanent and valuable message about the counterpart’s emotions. Therefore, we conclude that attachment avoidance is manifested by a stable scanning pattern, in which initial interest in all emotional cues shifts to gaze directed at areas of comfort, represented by the upper face or by emotionally neutral and irrelevant regions.

In the context of situational factors influencing gaze behavior with respect to high emotional and cognitive demands, such as truthful and deceptive communication, gaze behavior appears to be influenced by both cognitive and interpersonal demands [6,12,13,14,15,16]. Contrary to traditional assumptions that associate deception with gaze aversion [36], recent findings indicate that deceivers often increase their visual attention to the interlocutor’s face, especially the eye-region, to enhance perceived credibility and monitor the interlocutor’s responses [6,37,38].

This strategic gaze behavior is further supported by evidence that deceivers tend to divert their attention from task-relevant stimuli and focus on cues that facilitate impression management [39]. Individuals who feel confident and are motivated to appear credible are more likely to direct their gaze toward socially salient facial regions, such as upper face, rather than toward peripheral or non-social cues [6]. Additionally, when individuals have time to prepare their responses, they can better regulate nonverbal cues, enabling more effective impression management [6,16]. In contrast, spontaneous responses, particularly under high cognitive load, often result in greater visual disengagement [10,40], consistent with findings that reduced visual engagement minimizes distraction [4,41] and supports internal cognitive operations such as memory retrieval and problem solving [9].

Taken together, these findings highlight the importance of examining how stable individual differences, such as attachment avoidance, interact with situational factors that place high emotional and cognitive demands on individuals, such as deceptive communication. Although gaze behavior has been shown to reflect both regulatory strategies associated with attachment avoidance [19,25,31,32,33,34,35] and strategic management of both cognitive and interpersonal demands [6,12,13,14,15,16], these perspectives have developed in parallel. Consequently, the intersection of personality-based mechanisms and situationally induced demands in shaping gaze patterns remains unexplored. Bridging this gap may provide a more nuanced understanding of the functional significance of gaze behavior in communication. In particular, integration of personality-based and situational perspectives may extend the application of attachment theory to the study of emotion recognition processes and interpersonal dynamics. Additionally, including specific situational contexts, such as deceptive communication, may advance theoretical frameworks concerned with behavioral regulation under conditions of cognitive and emotional load.

## 3. The Present Study

Although gaze behavior has been extensively studied as a situational cue of inner state of mind [6,12,42,43,44] and as a result of individual differences grounded in personality theories during interactions [19,30,31,35], to our knowledge, no previous work has systematically examined the combined role of these two sources in shaping gaze scanning patterns. To address this gap, the present study draws on two well-established research domains that have independently contributed to our understanding of gaze behavior: (1) theoretical and empirical findings from attachment theory, which explain behavior during interactions and the stable scanning patterns of individuals with an avoidant orientation; (2) research findings on changes in gaze behavior resulting from situationally induced high cognitive and interpersonal demands, in which specific gaze behavior serves a regulatory function. Building on these foundations, the present study aims to experimentally examine whether individuals, in relation to their avoidant attachment orientation, display different gaze directions toward three regions of interest depending on situational demands, specifically when engaged in truthful versus deceptive communication towards a counterpart.

Based on previous studies examining situational effects of deceptive and rehearsed communication, the basic dynamics indicate that more attention is paid to the upper face region during rehearsed communication than during spontaneous communication with the counterpart [6]. Similarly, deceptive communication has been associated with increased gaze toward the upper face compared to truthful communication [6,37,38]. These findings suggest that prepared deception should elicit the highest level of visual focus on the interlocutor’s eyes. In this context, the current study will examine how attachment avoidance moderates these gaze dynamics, specifically focusing on the following: Avoidantly attached individuals tend to divert attention from relational and positive emotional cues, such as faces [30,31], and instead focus on neutral or non-social stimuli [19] or attend to the upper face region [19] associated with negative emotions [3]. This pattern reflects a regulatory strategy intended to maintain emotional distance, minimize affective engagement, and preserve a perceived zone of psychological safety [34]. Consistent with the vigilance–avoidance hypothesis [31,45] and the concept of the area of comfort [1], they may initially attend to socially salient cues but quickly disengage from emotional signals to decrease relational risks and downregulate arousal. These attentional patterns are expected to be evident in emotionally charged contexts, such as spontaneous deception, spontaneous truth-telling, or small talk, where demands on emotional regulation are high. In contrast, structured conditions like rehearsed responding may not activate the attachment system, resulting in more context-sensitive gaze behavior. Therefore, we hypothesized the following, while working with three regions of interest (upper face, lower face, and background):

- (H1) Higher attachment avoidance is generally associated with increased fixations on the background and upper face (i.e., eye region) and decreased fixations on the lower face.
- (H2) Considering the specific situational conditions of emotionally charged contexts (such as small talk, rehearsed and spontaneous truth and lie responding), attachment avoidance moderates the relationship between condition and gaze fixations, resulting in increased attention to the upper face (i.e., eyes) and background only in spontaneous scenarios and small talk, but not in rehearsed scenarios.

Together, H1 and H2 indicate that attachment avoidance would interact with situational communicative demands resulting in a specific pattern of scanning characterized by generally higher attention to the upper face and background regions, and altering the general pattern of focus on the upper face (i.e., eyes) area and background, especially during spontaneous conditions and small talk.

## 4. Materials and Methods

### 4.1. Participants and Ethics

Participants were recruited from the general population using a non-probability convenience sampling strategy, with registration facilitated through social media (e.g., Instagram, LinkedIn) and QR-coded posters in public areas such as bus stations and university corridors. Data collection took place from 28 June 2023, to 4 October 2024, reflecting the logistical demands of the within-subject experimental design. The total sample consisted of 55 participants, of whom 61.82% (*n* = 34) were women aged from 18 to 35 years (*M* = 21.12 years, *SD* = 3.81 years) and 38.18% (*n* = 21) were men aged from 18 to 35 years (*M* = 22.57 years, *SD* = 5.20 years). A subset of participants produced corrupted eye-tracking data, primarily due to pronounced head movements, eye occlusion, or related artifacts, resulting in the exclusion of 14 individuals for excessive missing data. Additionally, 10 participants did not complete the ECR questionnaire. Thus, the final sample included 31 participants: 61.29% (*n* = 19) were women aged 18 to 28 years (*M* = 20.79, *SD* = 2.63), and 38.71% (*n* = 12) were men aged 18 to 35 years (*M* = 22.92, *SD* = 4.95).

All participants have been properly instructed and have indicated that they consent to participate by signing the written informed consent form. The experiment was conducted in accordance with the Ethical Principles for Psychologists and the Code of Conduct of the American Psychological Association [46]. The project was approved by the Palacký University Institutional Review Board (IRB): FF UP Ethics Panel for Research under the reference number 03/2023.

### 4.2. Methods and Conditions

#### 4.2.1. Experiences in Close Relationships

Attachment avoidance was assessed using the Czech adaptation of the Experiences in Close Relationships questionnaire [47]. This 36-item measure evaluates two dimensions of adult attachment: Attachment Anxiety (ANX) and Attachment Avoidance (AVO). Each dimension includes 18 items rated on a 7-point Likert scale from 1 (strongly disagree) to 7 (strongly agree). In the current study, both subscales showed high internal consistency (α = 0.834 for avoidance; α = 0.876 for anxiety). For the present analyses, only the AVO subscale was used.

#### 4.2.2. Eye-Tracker Tobii Pro Spectrum

Eye fixations were recorded using the Tobii Pro Spectrum eye-tracker, which sampled at 300 Hz. According to the manufacturer’s specifications, the eye-tracker provides an RMS precision of 0.01° and an accuracy of 0.3° under optimal conditions. The Scene Camera mode of the Tobii Pro Spectrum was used, so the stimulus was not displayed on a screen, allowing a real person (the interviewer) to sit in front of the participant. The eye tracker was placed on a table approximately 65 cm from the participant, ensuring the eyes remained centered within the recording area. Participants were not required to use head-stabilization equipment.

A binocular calibration procedure required participants to follow four points in the laboratory to calibrate both eyes. If the final deviation exceeded 0.5° for either eye, recalibration was performed (required for only one participant). The average calibration accuracy was 0.416 degrees, the average calibration precision SD was 0.234 degrees, and the calibration precision RMS was 0.223 degrees.

**Figure 2:**
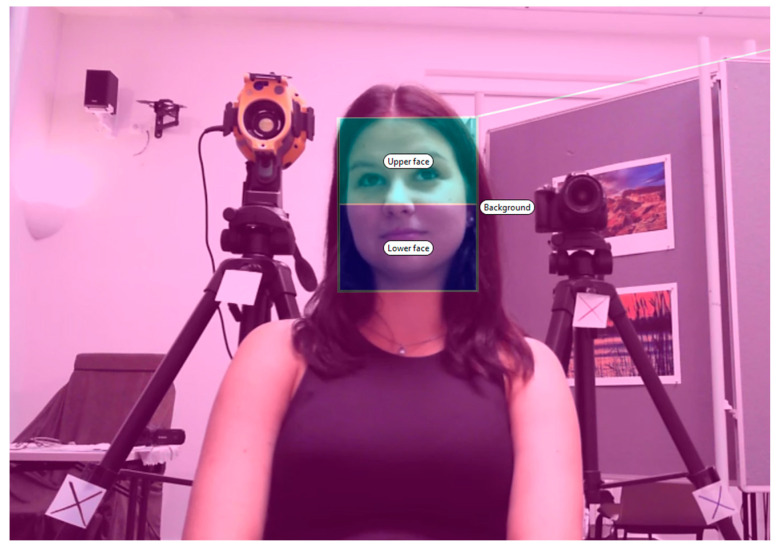
Used ROIs for the eye-tracking analyses.

Because head-boxed, scene-camera-based setups without screens may be susceptible to parallax error when the plane of regard (i.e., the depth plane of the fixated object) does not coincide with the calibrated plane [48], we designed the setup to minimize depth-related discrepancies. Calibration points were positioned at a comparable viewing distance to the interviewer’s face and only slightly behind the interviewer (see calibration crosses in Figure 2), thereby reducing potential misalignment between the calibrated plane and the primary plane of regard. In addition, participants predominantly fixated on the interviewer, and the interaction did not involve frequent switching between objects at markedly different depths. Given the relatively large ROIs used in subsequent analyses, any residual parallax-related spatial displacement is unlikely to have affected ROI assignment. Nevertheless, parallax effects cannot be entirely excluded and are acknowledged as a methodological limitation.

All eye-tracking data were processed and analyzed with Tobii Pro Lab v1.194 software.

#### 4.2.3. Small-Talk Condition

Prior to the main interview, participants took part in a brief small-talk session that served as a separate baseline condition. The interviewer asked a standardized set of informal questions (e.g., “How do you feel?”, “Did you find the building easily?”, “How did you hear about the experiment?”). This phase was designed to help participants relax, establish rapport, and collect baseline behavioral data in a neutral conversational context.

#### 4.2.4. Rehearsed Truth and Lie Conditions

In the rehearsed condition, participants received two general conversational questions about work and study life designed to elicit one truthful and one deceptive response. They had seven days to prepare their answers, following the procedure established in previous research [6]. Participants could choose how much time to spend preparing during this period but were not allowed to use written notes during the experimental session.

#### 4.2.5. Spontaneous Truth and Lie Conditions

In the spontaneous condition, participants completed two preliminary tasks approximately one hour before the interview. First, they visited four predetermined locations and photographed specified landmarks or objects. Next, they returned to the laboratory and spent 15–20 min assembling simple puzzles that, when completed, revealed a short narrative. Neither preparatory activity was recorded. After these tasks, participants sat in front of the recording equipment for a structured interview. They answered two questions about the preceding activities, one requiring a truthful response and one a deceptive response. The order of truthful and deceptive responses was counterbalanced across participants. Instructions indicating which question to answer truthfully or deceptively were provided in a sealed envelope placed in front of each participant. This procedure, adapted from previous studies [49,50,51], ensured that truthful answers referred to recent, personally experienced events, thereby minimizing the risk of memory decay or confabulation.

### 4.3. Procedure and Settings

**Figure 1:**
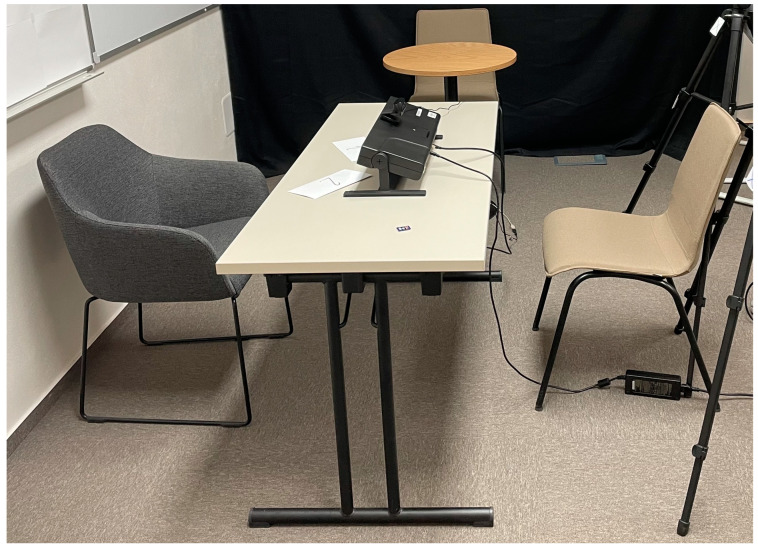
Laboratory setting.

The experiment was conducted in a 35 m^2^ laboratory with a room temperature set on 20.5 °C. The eye tracker was positioned at the center of the room, directly opposite the participant, and adjusted in height to capture the entire face as depicted in Figure 1.

Seven days before the experiment, participants received instructions for the rehearsed scenario and an online link to complete the ECR questionnaire along with demographic information. On the day of testing, they first signed an informed consent form and completed two preliminary tasks outside the laboratory as it is described above. These tasks were not recorded.

After the preliminary tasks, participants entered the laboratory and were seated in front of the recording devices for eye-tracker calibration. Once calibration was complete, the experimenter activated the device and left the room.

The interview then began. All interviewers were female (*N* = 4; age 24–28 years) to maintain standardization and reduce variability related to gender dynamics. Each interviewer was trained to conduct the interview using a printed script with fixed wording and question order to ensure consistency.

The interview started with a brief small-talk segment, serving as a baseline condition. Participants were then questioned under two types of conditions: (1) rehearsed; (2) spontaneous. In both conditions, participants were instructed to respond truthfully in one case and deceptively in another. The assignment of truthful and deceptive responses was counterbalanced across participants. Instructions indicating which spontaneous question to answer truthfully or deceptively were provided in a sealed envelope placed in front of each participant. The interviewer remained blind to all of the participants’ instructions. Eye fixations were continuously recorded during all phases of the interview, including small talk, rehearsed, and spontaneous conditions, under both truthful and deceptive responses. This design allowed systematic comparison of gaze behavior across different communicative contexts. To increase motivation to deceive [52], participants were told that the most convincing liar, determined by whether the interviewer believed their answers, could win a monetary reward (€158).

After the interview, participants completed a questionnaire indicating which of their responses were truthful and which were deceptive, providing ground-truth labels for data validation. Finally, each participant took part in a debriefing session lasting 5–30 min.

### 4.4. Data Pre-Processing

Tobii Pro Lab software was used to analyze participants’ eye fixations on the ROIs defined on the interviewer’s face (see Figure 2). The present study used 31 dynamic eye-tracking scene-camera recordings, each corresponding to one participant, capturing gaze behavior throughout the live interaction.

We defined the upper face (including the forehead, eyes, and upper half of the nose) and lower face (including the lower half of the nose, mouth, and chin) so that both regions maintained approximately equal surface area to ensure balanced statistical weighting. Technical constraints regarding the recording camera required us to work with two different video resolutions (960 × 540 px and 1920 × 1080 px). Therefore, ROI dimensions are reported in millimeters (with pixel values in parentheses) using the calibration plane scaling (528 × 297 mm). The average ROI dimensions (width × height) for the lower and upper face ROIs were approximately 74.25 × 46.20 mm (135 × 84 px) for the lower-resolution recordings (960 × 540 px) and 89.38 × 51.98 mm (325 × 189 px) for the higher-resolution recordings (1920 × 1080 px), varying slightly by interviewer to preserve relative anatomical proportions. Because interviewers were instructed to remain relatively static, we used an event-based manual adjustment protocol rather than continuous frame-by-frame tracking; ROI bounding boxes were repositioned manually only when the interviewer shifted posture to ensure consistent anatomical coverage. The background ROI consisted of the remaining visible area, strictly excluding the face.

To contextualize spatial data quality relative to the ROI geometry, calibration performance on the 528 × 297 mm plane indicated an average accuracy of 2.24 mm and precision of 3.22 mm (SD) (2.94 mm RMS). These errors are small compared with the facial ROI dimensions (approximately 74 × 46 mm in the lower-resolution recordings and 89 × 52 mm in the higher-resolution recordings), meaning that gaze-position uncertainty typically corresponds to only a few millimeters—i.e., a small fraction of ROI width/height. Therefore, the relatively large ROIs and the stable interviewer positioning reduced the likelihood that calibration-related spatial deviations would shift fixations across ROIs, although minor misclassification can still occur at ROI boundaries (e.g., between upper vs. lower face, or face vs. background), particularly when fixations fall close to the edge. We also acknowledge limitations related to manual ROI definition and resolution variability, which are addressed in detail in the Discussion section.

### 4.5. Statistical Analyzes

All statistical analyses were conducted in the R 4.4.1 environment. The following packages were used: lme4 [53], lmerTest [54], emmeans [55], car [56], performance [57], effectsize [58], tidyverse [59], and dplyr [60]. Given the hierarchical nature of the data, with repeated measures nested within participants, linear mixed-effects models (LMMs) were used. Instead of log-transforming fixation durations, fixation proportions were computed for each participant using the Formula (1):

Proportional data = (Fixation time on ROI)/(Total interval duration),(1)

The total interval duration was defined as the active stimulus window for each experimental condition (e.g., small talk, rehearsed truth), calculated from the onset to the offset of the video stimulus and excluding any pre- or post-interval fixation patterns. This approach was chosen due to the low fixation values observed, particularly for the upper-face ROI, which would have compromised model stability under a logarithmic transformation and required arbitrary constant adjustments. Proportional scaling normalized fixation durations relative to each participant’s total condition time and allowed model convergence with random intercepts. To directly test the operationalized hypotheses about the influence of attachment avoidance on gaze behavior, mean-centered attachment avoidance scores (ECR_AVO_c) from the ECR questionnaire were added to the model, along with their interactions with condition and ROI using the following Equation (2):

Fixation proportion ~ All conditions ∗ ROI ∗ ECR_AVO_c + (1|ID),(2)

Model assumptions including normality of residuals, absence of multicollinearity, homoscedasticity, and model convergence were verified using Q–Q plots, VIF scores from the performance package, and inspection of homoscedasticity plots. All categorical predictors were treated as factors. Contrast coding was set to contr.sum to allow for valid Type III ANOVA tests. Effect sizes were calculated using partial eta-squared ($\eta_{p}^{2}$) using following Formula (3) [61,62]:

(3) $$\eta_{p}^{2}\;=\;(F\;\times \;{df}_{\mathrm{effect}})/(F\;\times \;{df}_{\mathrm{effect}}\;+\;{df}_{\mathrm{error}}),$$

- *F*: F-value of the effect;
- *df*_effect_: degrees of freedom of the effect;
- *df*_error_: degrees of freedom of the error.

Degrees of freedom were estimated using the Satterthwaite approximation [63]. Post hoc pairwise comparisons were conducted with the emmeans package [55] applying Bonferroni corrections to adjust for multiple comparisons.

## 5. Results

**Figure 3:**
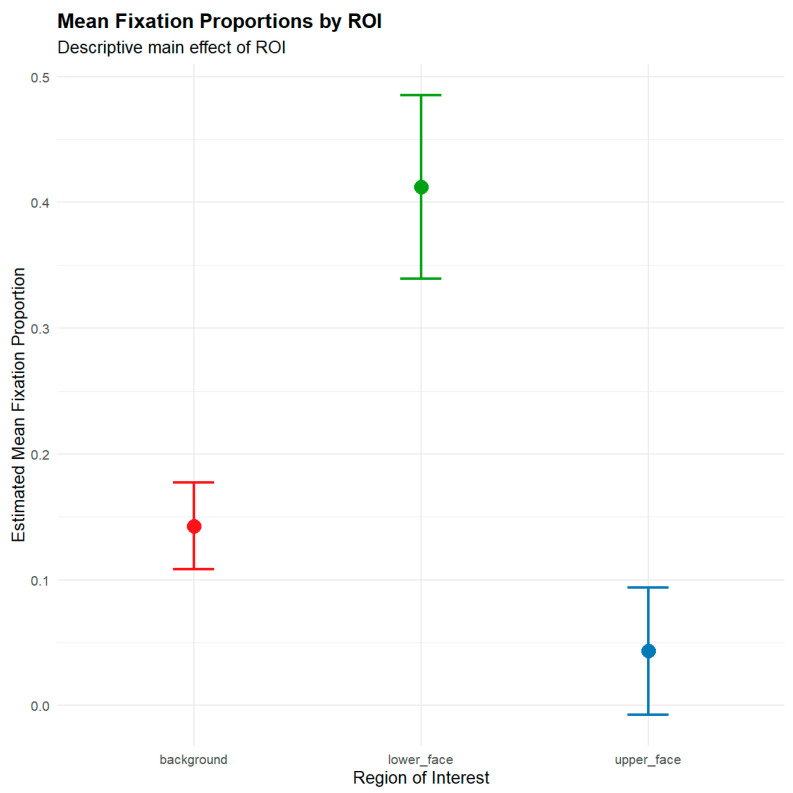
Mean Fixations Proportions by ROI.

A significant main effect of ROI, *F*(2, 321.65) = 135.28, *p* < 0.001, $\eta_{p}^{2}$ = 0.66, indicating that fixation proportions differed significantly across facial regions, was found. As shown in Figure 3, participants spent the highest proportion of fixations on the lower face (*M* = 0.41, *SE* = 0.03), followed by the background (*M* = 0.14, *SE* = 0.01), and the least on the upper face (*M* = 0.04, *SE* = 0.02).

A significant ROI × attachment avoidance interaction, *F*(2, 322.41) = 10.58, *p* < 0.001, $\eta_{p}^{2}$ = 0.06 was found, consistent with the Hypothesis 1. In contrast, the main effect of attachment avoidance was not significant, *F*(1, 31.73) = 0.07, *p* = 0.790, $\eta_{p}^{2}$ < 0.01.

**Figure 4:**
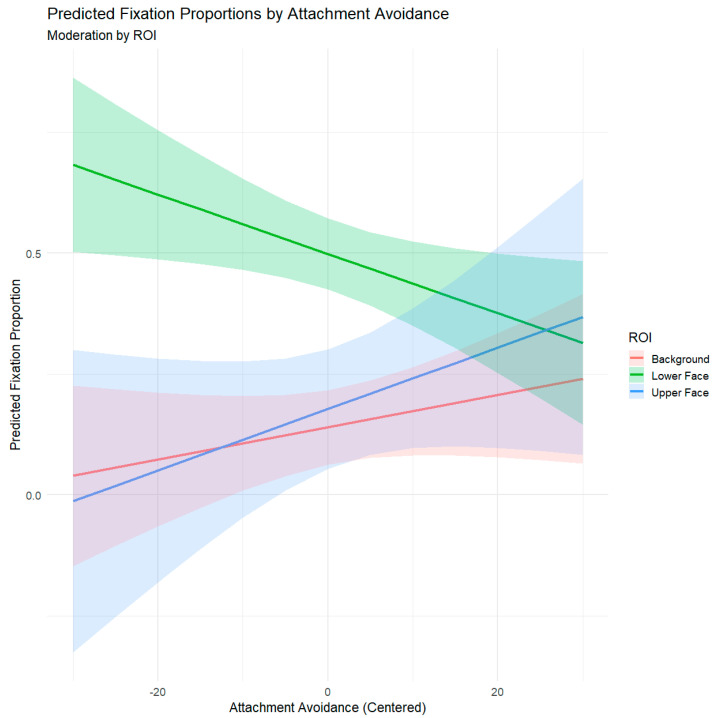
Predicted Fixation Proportions by Attachment Avoidance and Moderation with ROI. Note: Positive values indicate higher attachment avoidance, and negative values indicate lower attachment avoidance.

Estimated marginal trends clarified the nature of the ROI × avoidance interaction. Consistent with Hypothesis 1, higher attachment avoidance was significantly associated with fewer fixations on the lower face, *b* = −0.00381, *SE* = 0.00125, *95% CI* [−0.00630, −0.00132], suggesting decreased attention to the part of emotionally expressive cues. Although increased fixations on the upper face (*b* = 0.00084, *SE* = 0.00170, *95% CI* [−0.00252, 0.00421]) and background (*b* = 0.00211, *SE* = 0.00127, *95% CI* [−0.00044, 0.00465]) were not statistically significant, the positive visual trends are directionally consistent with theoretical predictions, indicating potential tendencies toward increased monitoring of the upper face region (i.e., eyes) and visual disengagement from the social stimulus (i.e., the lower face) by looking at the background. Predicted fixation trajectories across ROIs as a function of attachment avoidance are shown in Figure 4.

Contrary to Hypothesis 2, no significant interactions between attachment avoidance and conditions were found. Specifically, the two-way interaction between Condition and Attachment Avoidance was not significant, *F*(4, 316.30) = 0.40, *p* = 0.807, and the three-way interaction (ROI × Condition × Attachment Avoidance) also was not significant, *F*(8, 314.83) = 0.35, *p* = 0.945. These results indicate that the effects of avoidance on gaze allocation did not differ across conversational contexts.

## 6. Discussion

This study offers new insight into gaze behavior during interpersonal communication, emphasizing the influence of attachment avoidance on attention to facial regions. By integrating individual differences grounded in personality theory with situational demands, the primary aim of this study was to experimentally examine whether individuals show, with respect to an avoidant attachment orientation, different gaze directions toward regions of interest depending on situational demands, specifically when engaged in truthful versus deceptive communication towards a counterpart.

Through all analyses, a strong main effect of ROI, with participants fixating longer on the lower face than on the upper face or the background was found. Additionally, some participants showed little to no fixations in the interviewer’s upper face region. To rule out an ROI assignment artifact, we visually validated fixation locations by overlaying fixations on scene camera images in Tobii Pro Lab (snapshot projection). In multiple cases, background-classified fixations were clearly outside the face ROIs and not concentrated near ROI borders, indicating that this pattern reflects genuine gaze behavior rather than misclassification. This result aligns with earlier research suggesting that the mouth region plays a key role in speech perception and conversational engagement by providing visually informative cues [64,65,66]. Focusing on the mouth area may reflect a socially adaptive strategy that allows listeners to remain attentive [67].

A central contribution of this study is evidence that attachment avoidance systematically influences gaze allocation across facial regions. Although no main effect of avoidance emerged, the significant ROI × avoidance interaction (see Figure 4) supports our hypothesis that higher levels of attachment avoidance are associated with reduced fixation on the lower face. In contrast, non-significant but directionally consistent trends for the upper face and background suggest a modest increase in fixation among more avoidant individuals, reflecting a tendency to disengage from positive emotionally expressive cues while maintaining attention to the upper face or neutral areas. This pattern aligns with the broader literature on avoidance as a deactivating regulatory strategy that limits engagement with positive, emotionally charged or relational cues [26,27,34]. Therefore, attachment avoidance alters face-scanning patterns in an interaction, regardless of situational demands.

Additionally, these findings are in line with the vigilance–avoidance hypothesis, which proposes a two-stage attentional sequence characterized by initial orientation toward emotionally salient cues, followed by perceptual withdrawal as a means of emotion regulation [31,35]. Although our paradigm did not capture temporal dynamics directly, the observed allocation pattern suggests that avoidant individuals may be prone to increased gaze fluctuation across ROIs. This variable gaze pattern may reflect previously conceptualized gaze scanning behaviors, such as strategic monitoring of an interlocutor’s affective signals [35], use of “areas of comfort” [1,19], or a moderate “perceptual escape” strategy [10] to reduce arousal and preserve psychological autonomy during socially demanding interactions [30,31].

The stability of our findings across all interview conditions (i.e., small talk, rehearsed truth and lie, spontaneous truth and lie) suggests that avoidance-related gaze tendencies reflect enduring attentional traits rather than situationally induced behaviors. These results extend previous research by demonstrating that gaze behavior in interpersonal contexts cannot be understood solely through situational factors such as truth versus lie or spontaneous versus rehearsed responding. Instead, attachment avoidance exerts a trait-level influence on visual attention, stabilizing gaze allocation and potentially masking situation-related effects. While this stability may serve emotional regulation, it could also limit interpersonal attunement by reducing attention to facial regions that convey affective and communicative cues. Future research should address two important challenges in this area. First, it remains to be tested whether the dominant role of attachment avoidance holds across different situational conditions, including other types of situational cues, especially relational stimuli between acquainted individuals, and how the dominance of the scanning pattern characteristic of avoidant attachment unfolds during interaction, with a more fine-grained differentiation of ROIs. Second, an important direction for future research is to examine how the scanning pattern associated with attachment avoidance influences emotion recognition and the quality of interpersonal interaction [22].

Although this study provided new insight into gaze behavior during interaction from an integrative perspective by jointly considering the influence of situational factors and stable individual differences such as attachment avoidance, several limitations should be acknowledged.

First, the spontaneous condition, which required participants to recall real-life experiences from an unfamiliar environment, may have unintentionally increased cognitive load [68,69], thereby reducing the distinction between varying cognitive load conditions. Future studies could address this by incorporating narrative-support techniques, such as sketching during recall [49,51,70], or by manipulating environmental familiarity to better isolate contextual effects.

Second, manual extraction of ROIs in dynamic eye-tracking recordings may have introduced coding variability and bias. Unlike static paradigms with fixed stimuli [6], our design required, in case of the interviewer movement, frame-by-frame manual placement, increasing the likelihood of inconsistencies. This observer-dependent variability may have affected the precision of fixation measures and reduced comparability across participants. Although the study used a design based on real interaction, which is closer to natural dyadic communication, new challenges have arisen for data preprocessing. Therefore, future research should employ specific, validated automated ROI detection methods designed for dynamic face-to-face interactions, such as the workflows and face-mapping techniques [21,22,48] to ensure more consistent and reproducible mapping of facial regions. Additionally, technical constraints required the use of two different video resolutions (960 × 540 px and 1920 × 1080 px), necessitating proportional scaling of ROI dimensions. While relative anatomical proportions were preserved, this variability in pixel density represents a methodological limitation that should be acknowledged.

Moreover, the present analysis relied exclusively on static fixation proportions, offering a useful but limited snapshot of attentional allocation. This approach does not account for the temporal dynamics of gaze behavior, potentially overlooking meaningful patterns such as shifts between ROIs, sequential dependencies, or saccadic transitions that occur over time. Future research could benefit from incorporating temporal modeling techniques, such as Long Short-Term Memory (LSTM) [71] or hidden Markov Models [72], to capture the evolving structure of gaze patterns and provide a more comprehensive understanding of attentional processes. In addition, future studies could consider using eye-tracking glasses instead of a stationary eye tracker, which would enable the integration of automatic scene-segmentation tools, such as the method introduced by Niehorster et al. [73]. Including additional ocular metrics, such as pupil dilation, could also improve sensitivity to cognitive and emotional processes [39,42,74].

Last but not least, future work should expand the statistical framework of deception research by comparing different modeling approaches, including linear mixed-effects models and signal detection theory within generalized mixed models, to distinguish response bias from genuine discriminability and strengthen analytical robustness [75]. Person-centered analytic approaches, such as cluster or latent profile analyses, could reveal distinct gaze phenotypes associated with different combinations of attachment styles and cognitive control.

## 7. Conclusions

In conclusion, this study addressed a gap in the literature by demonstrating that attachment avoidance systematically shapes gaze behavior across different communicative demands. While previous research typically examined situational and dispositional influences separately, our findings showed that individuals exhibit stable visual attention patterns related to their attachment avoidance, characterized by reduced engagement with lower-face regions regardless of interaction context. In this vein, this work bridges two previously distinct research streams: the situational and dispositional determinants of gaze behavior. These insights advance the theoretical foundations of gaze research and provide a basis for future studies to further investigate the dynamic interplay between personality and context in interactive communicative settings.

## Data Availability

The data presented in the study are openly available in OSF at https://osf.io/e673k/overview?view_only=e2ac5d83bbc1471cb2443277884aba59 (accessed on 29 December 2025).

## Abbreviations

The following abbreviations are used in this manuscript:

ROI	Region of interest
LMMs	Linear mixed-effects models
ECR	Experiences in Close Relationships questionnaire
AVO	Attachment Avoidance
ANX	Attachment Anxiety
ECR_AVO	Attachment Avoidance measured via Experiences in Close Relationships questionnaire
RMS	Root Mean Square
LSTM	Long Short-Term Memory
